# Resveratrol and Wine: An Overview of Thirty Years in the Digital News

**DOI:** 10.3390/ijerph192315815

**Published:** 2022-11-28

**Authors:** Paula Silva, María P. Portillo, Alfredo Fernández-Quintela

**Affiliations:** 1 Laboratory of Histology and Embryology, Institute of Biomedical Sciences Abel Salazar (ICBAS), Rua de Jorge Viterbo Ferreira 228, 4050-313 Porto, Portugal; 2 ICNOVA—NOVA Institute of Communication, NOVA School of Social Sciences and Humanities, Universidade NOVA de Lisboa, 1069-061 Lisbon, Portugal; 3 Nutrition and Obesity Group, Department of Nutrition and Food Science, University of the Basque Country (UPV/EHU), 01006 Vitoria-Gasteiz, Spain; 4 Lucio Lascaray Research Institute, 01006 Vitoria-Gasteiz, Spain; 5 BIOARABA Health Research Institute, 01006 Vitoria-Gasteiz, Spain; 6 CIBERobn Physiopathology of Obesity and Nutrition, Institute of Health Carlos III, 01006 Vitoria-Gasteiz, Spain

**Keywords:** resveratrol, wine, health communication, health literacy

## Abstract

Background: Resveratrol’s health benefits have received wide media coverage. Since resveratrol is usually associated with wine, informative texts about it should be prepared very carefully, since inaccurate website content could easily change people’s wine consumption behavior. This study aimed to assess the quality of informative texts related to resveratrol on science journalism websites. Methods: We analyzed 125 resveratrol posts on Science Daily, WebMD, and EurekAlert! published between 1990 and 2020. Results: A higher number of posts was published in the years in which the number of people looking for information on the internet also increased. The increase can also be related to David Sinclair’s notoriety, a fact that we called the “Sinclair effect”. Most of the posts are replications of universities’ press releases, mainly reporting resveratrol’s health benefits, which resulted from preclinical studies and cannot be translated to humans. Most of them mention wine in the text and some in the title. Conclusions: Wine is usually mentioned in headline resveratrol news, which could potentially influence wine consumption behavior. Scientists must intensify their efforts to communicate with the public to increase people’s health literacy. Online news portals should have science journalists skilled in exploring scientific data and their translation into a simple and accurate language.

## 1. Introduction

Media are a vital element in societies and democracies and are considered by many as the fourth power, after the legislative, executive, and judicial ones [1]. The media’s function is to inform in a clear, precise, and objective way to increase population literacy. People’s thoughts and attitudes are strongly influenced by the media. Therefore, media assume an undeniable pedagogical role, as they are an important factor in individual decisions [2]. Scientific journalism contributes to the process of scientific instruction, bringing citizens closer to the universe of science and technology and, consequently, has a pedagogical and complementary role to that of education [3]. To be successful in that pedagogical function, science journalism must establish a bridge between science and journalism speeches that have different goals and audiences. For a long time, scientists had difficulty dialoguing with journalists, because scientists are familiar with scientific methods, rules, and dissertations, which are different from the journalist’s language [4]. Journalists are essentially oriented towards more simplified approaches and give primacy to the public interest, controversy, and novelty [4,5].

With technological advancement, the number of people looking for information on the internet has significantly increased [6]. The internet is a large network that provides a lot of information and whose consumption is controlled by the reader, in the sense that each person seeks the information, chooses the reading depth (following the links provided for more detailed reading), and chooses the interaction (comments, shares, forums…). This growing internet engagement has led to the rise of online journalism [7]. Science journalists are increasingly present on the internet, where they produce content in different formats and disseminate it in various ways. Science journalists must reduce the complexity and ambiguity of scientific results before divulging them to the public [8]. In this process, journalists usually seek to remove uncertainty from the speech and emphasize certainties about future expectations of scientific discovery. However, this is not always easy or possible, especially for complex, controversial scientific issues that may be of economic interest [9]. Online news websites are the most common means to spread the news and make claims about health and millions of people use them to obtain new information [10]. However, misleading claims are common [11,12] and may have adverse consequences for public health and create confusion and suspicion [12,13]. This can be due to, among other things, misleading titles, causality affirmations not supported by data, magnifying the significance of the research, inappropriate extrapolation of results, and minimizing or ignoring study limitations [11,14,15,16].

Resveratrol was first isolated in 1940 from white hellebore (*Veratrum grandiflorum* O. Loes) [17]. In 1963, resveratrol was identified in fallopia japonica (*Polygonum cuspidatum*), a plant used in traditional Chinese and Japanese medicine and one of the main sources of resveratrol [18,19]. Langcake and Pryce (1976) [20] first detected the presence of resveratrol in grapes (*Vitis vinifera*) and suggested that resveratrol was synthesized in response to external stimuli, such as exposure to fungi (e.g., *Botrytis cinerea*) or ultraviolet light. Only in the 1990s was resveratrol identified in red wine [21]. Since then, several benefits of drinking wine, because of its resveratrol content, have been promoted. However, for many people, the act of consuming wine is still shrouded in insecurity, because, in addition to the benefits, it appears to be associated with a series of risks. Consumers are aware that alcohol abuse/addiction results in damage to multiple organs. Different etiologies may be involved in injuries caused by chronic consumption of alcoholic beverages; most of these are associated with oxidative stress and inflammatory response activation, usually promoted by ethanol [22]. On the other hand, in 1991, a study known as the French Paradox, which was published in the Lancet in 1992, suggested that red wine consumption reduces the risk of heart disease [23]. Since then, consumers have been continually informed by the media about scientific studies that demonstrate the beneficial effects of moderate consumption of red wine. The antioxidant and anti-inflammatory effects of red wine associated with its moderate consumption have been demonstrated through epidemiological studies [24]. In addition to the importance of moderate consumption of red wine for heart disease protection, many studies have been published proving an equally beneficial effect concerning cancer, diabetes mellitus, inflammatory, neurodegenerative, kidney diseases, and as an aging delay [25,26,27,28,29]. The double effect on wine consumption raises the question of whether it can be considered a healthy and safe beverage.

To find published research on news coverage of wine and news media roles concerning health issues, we carried out searches, using mainly combinations of the keywords ‘wine’ and ‘news’ in the Scopus and ISI Web of Science databases. Surprisingly, no research has examined how scientific messages about wine are communicated through day-to-day news coverage, especially considering health issues. As previously mentioned, nowadays, it is difficult to conceive exclusively offline journalism. Moreover, as more and more people become active new media users, the increase in online news consumption is a reality. Furthermore, most online news consumers only skim the news, verifying headlines occasionally rather than engaging at a certain time of day to read the news [30], which emphasizes the importance of looking at news headlines of online news. Considering the wine controversy health effects and that we are investigating the biological effects of resveratrol, as a first approach, we decided to assess the quality of informative texts related to resveratrol on science journalism websites and evaluate if wine is used as a strategy for drawing attention and capturing readers’ curiosity. In this paper, we first describe the methodology used to analyze resveratrol posts on Science Daily, WebMD, and EurekAlert! published between 1990 and 2020; then, we describe and discuss the results obtained. A special section was included to discuss the news that was not supported by resveratrol research.

Science communication in online news represents an expanding area of research that is yet to be fully characterized. This is the first study to evaluate news coverage of resveratrol in online news. We intended to identify information not supported by research results and if the wine was used in a misleading way, which could potentially influence a reader’s health-related behavior. Our results seem to indicate shortcomings in online news and/or press releases describing resveratrol research. Some online news had misleading titles, commonly using wine to increase its impact. The major limitation of this work is that it is a qualitative analysis of online news content. In the future, the triangulation method using other information sources must be applied. In conclusion, our results indicate that it is important to avoid the use of attractive headlines in online news and press releases because they have the potential to mislead patients, other scientists, and the public. The evidence also indicates that this change to press releases is likely to carry through into the news. Therefore, both journalists and researchers should realize that media-generated health literature may misinform people. This could be one major part of the challenge to increase awareness and decrease confusion about health and lifestyle choice, including the decision about wine consumption.

## 2. Materials and Methods

Since resveratrol is naturally associated with the consumption of wine, health effects of which are controversial, we aimed to compare what has been published about resveratrol in the digital news with the existing scientific evidence.

We looked for posts that report scientific results related to the resveratrol effect on human health. We excluded skin benefits because we aimed to analyze the effect of resveratrol oral consumption, which is in line with wine intake. We looked for posts written and published between 1990 and 2020, to cover the 30 years since the French Paradox study was published.

First, we carried out a preliminary search on Google News, using resveratrol as a search term, to find the online news portals with more posts published in that period. We found that Science Daily, WebMD, and EurekAlert! were the main contributors which published 22%, 15%, and 30%, respectively. Therefore, we decided to search for resveratrol news in those websites. Science Daily is an American website launched in 1995 that aggregates press releases and publishes lightly edited press releases about science. EurekAlert! was established one year later (1996) and is a nonprofit news-release distribution platform operated by the American Association for the Advancement of Science (AAAS) as a resource for journalists and the public. EurekAlert! hosts news releases produced by universities, journal publishers, medical centers, government agencies, corporations, and other organizations engaged in all disciplines of scientific research. These news releases may describe research findings recently published in peer-reviewed journals, timely information related to the business, innovation, and societal aspects of science and details of grants, awards and honors, books, and scientific meetings. Founded in 1998, WebMD is an American corporation known primarily as an online publisher of news and information about human health and well-being.

We used resveratrol as a search term and we only considered posts with both a defined date and author/source. We obtained 183 posts: 63% published in Science Daily; 20% published in WebMD; and 17% published in EurekAlert!. We considered eligible for this study all the posts in which resveratrol was the focus. We analyzed 79 posts from Science Daily, 28 from WebMD, and 18 from EurekAlert!, which represents approximately 0.5% of the documents retrieved by Scopus, using resveratrol as search term in the “Article title, Abstract and Keywords” search field and defining the same date range (1990–2020).

**Figure 1:**
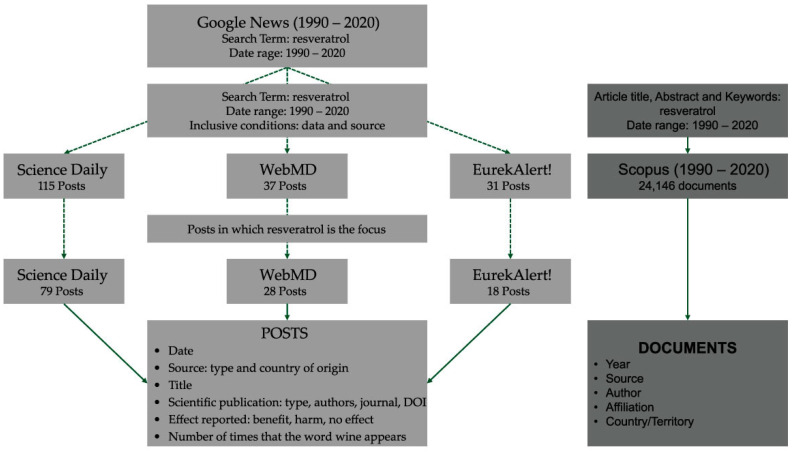
Methodology used in this study.

From each post we looked for the following information: date, source (type and country of origin), title, scientific publication (publication type, authors, source title, DOI), and type of effect reported (benefit, harm, no effect). We also registered the number of times that the word wine appears in the post. To compare the impact of this issue on digital news with the impact on the scientific sphere, we surveyed Scopus and used the “Analyze search results” feature which breaks up the results into seven categories from which we analyzed four: year, source, author, affiliation, and country/territory (Figure 1).

## 3. Results and Discussion

### 3.1. Resveratrol in the Digital News

**Figure 2:**
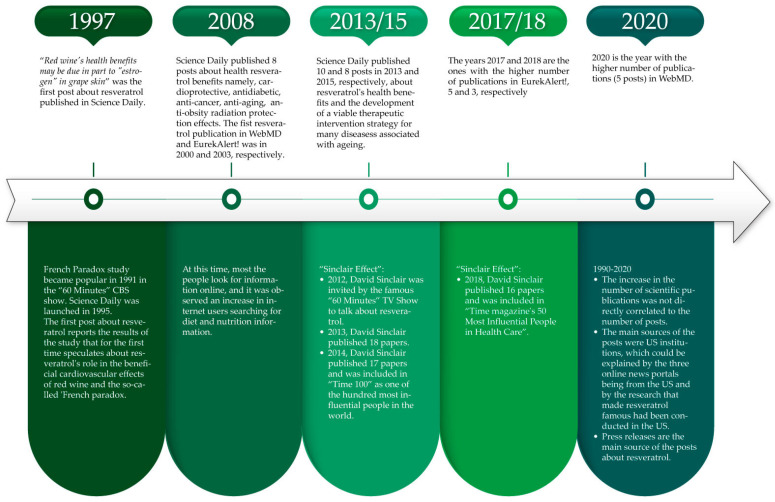
Resveratrol in the digital news.

The first post about resveratrol published in Science Daily dates back to 1997, the second one being posted six years later. After that, the only year without any publication is 2006. The first post published was “Red wine’s health benefits may be due in part to “estrogen” in grape skin” and summarizes the results of the paper by Gehm et al., 1997 [31], published in “*Proceedings of the National Academy of Sciences (PNAS)*” that, according to Scopus, was cited 935 times (Figure 2). The years that registered the higher number of publications are 2013 (10 posts) and 2008/2015 (8 posts). According to Scopus, there was no atypical increase in the number of publications in those years; therefore, the higher number of posts could be related to their topics. In 2008, all posts were about resveratrol health benefits, namely, cardioprotective [32,33], antidiabetic [34], anti-cancer [35,36], anti-aging [37], and radiation-protection [38] effects (Figure 2). The other post is about anti-obesity effects and summarizes the results of a study carried out by Pamela Fischer-Posovszky and presented at “The Endocrine Society’s 90th Annual Meeting” in San Francisco. According to Scopus, from those, the most cited paper is one written by Barger et al., 2008 [37] (510 citations) about the anti-aging effects of resveratrol. According to the paper of Michele Ybarra and Michael Suman [39] published in 2008, at that time, 7 in 10 Americans were online, more than half of whom were Internet health-information seekers. Suzanne Suggs and Chris McIntyre carried out a literature review about online health communication that was published in 2009 [40] and found that, at that time, approximately half of Internet users had searched for diet and nutrition information. These data could explain the higher number of Science Daily posts verified in 2008. Regarding 2013 and 2015, the higher number of posts could be associated to what we call the “Sinclair Effect” (Figure 2). The years in which David Sinclair published more papers were 2013 and 2014 with 18 and 17, respectively. In 2013, he published his famous papers about the possible development of a viable therapeutic intervention strategy for many diseases associated with aging based on the allosteric activation of SIRT1 by sirtuin-activating compounds (STACs), such as resveratrol. These papers were published in journals, including *Cell* [41], *Nature Communications* [42] and *Science* [43]. As a result of their work, David Sinclair started to be a renowned person. On 29 July 2012, he was invited by the famous “60 Minutes” TV Show to explain the results obtained in the epidemiological study known as the French Paradox about resveratrol. In 2014, David Sinclair was included in “Time 100” as one of the hundred most influential people in the world. One year later, David Sinclair gave an important interview to “The Scientist” about his career and his efforts to obtain funding for his lab. The research of David Sinclair about resveratrol promoted an increase in the number of posts published about this issue online (Figure 2). Regarding WebMD, the first publication was in 2000 and 2020 is the year with the higher number of publications (five posts). EurekAlert! started to publish about resveratrol later than the other two online news portals, the first post being published in 2003 (Figure 2). The years 2017 and 2018 had a higher number of publications, five and three, respectively. Despite the fact that any post was about David Sinclair’s work, we can also speculate the higher number of posts in 2018 is also due to the “Sinclair Effect”. In fact, 2018 was the third year of his career with more papers (16 papers). In 2018, he was included in “Time magazine’s 50 Most Influential People in Health Care”. In the three decades, between 1990 and 2020, according to Scopus, the number of publications has been increasing. The increase in the number of scientific publications during those 30 years was not directly correlated to the number of posts (Figure 2). We can conclude that the evolution of scientific research in this research field, which is usually expressed by the number of papers published, is not considered by journalists.

Science Daily always reveals the source of its posts. Half of them (42 posts) are news releases provided mainly by American universities. The source of three posts is the Loyola University Health System and the following universities contributed with two posts each: Louisiana State University and Oregon State University; University Health Network; University of Alabama; and University of Missouri-Columbia. Professional associations (12 posts), medical institutions (8 posts), research institutions (6 posts), academic editors (5 posts), scientific journals (4 posts), and 2 posts are other information sources. The sources are mainly from the USA (60 posts), followed by the United Kingdom with 6 posts and Canada with 5. WebMD has its own science writers, so no source is indicated in the post. The writers that wrote more posts about resveratrol are Miranda Hitti (four posts), Jennifer Warne (three posts), Kelli Miller (two posts), and Salynn Boyles (two posts). The authorship of four posts is assigned as WebMD Editorial Contributor. Most of the WebMD posts also indicate the name of the reviewer. Louise Chang reviewed eight posts, Dan Brennan reviewed three posts, and Arefa Cassoobhoy and Brunilda Nazario reviewed two posts each. EurekAlert! also publishes news releases provided by universities (seven posts), research institutes (three posts), academic editors (three posts), scientific journals (three posts), and private companies (three posts). Oregon State University is the only one mentioned twice. Elsevier contributed two posts and Resveratrol Partners LLC, DBA Longevinex is the company mentioned as the source of three posts. These results are in accordance with the country productivity in papers about resveratrol research, which, according to Scopus, is the US (5570 papers); China (4999 papers); Italy (1970 papers); India (1345 papers); and Spain (1244 papers). Regarding author affiliation, the Scopus top five are the Ministry of Education China (332 papers); Inserm (256 papers); Consiglio Nazionale delle Ricerche (247 papers); Chinese Academy of Sciences (221); and Harvard Medical School (194). The three online news portals are from the US, which explains why the Chinese institutions, despite their productivity, are not the main sources. The spark triggering the increase in resveratrol research was the publication of its activator effect on sirtuins and the extension of lifespan, which were carried out in the US, which also may explain the interest of these American online portals (Figure 2).

**Figure 3:**
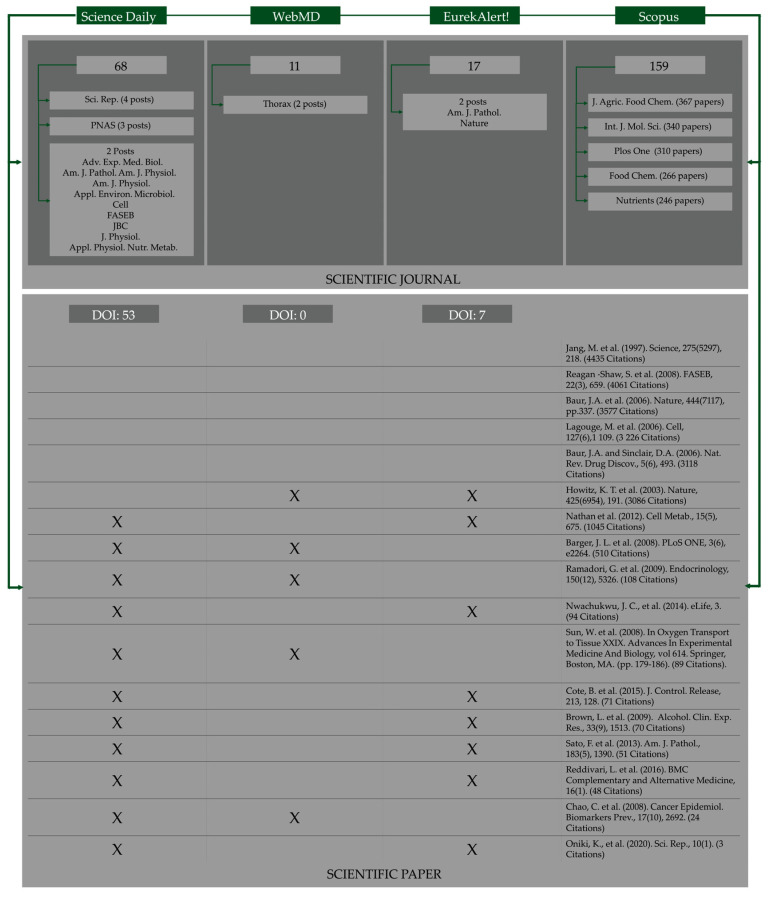
Information about the scientific publications provided by Science Daily, WebMD, and EurekAlert! and bibliometric data from Scopus retrieved in August 2022. *Rep.—Scientific Reports; PNAS—Proceedings of the National Academy of Sciences; Adv. Exp. Med. Biol.—Advances in Experimental Medicine and Biology; Am. J. Pathol. Am. J. Physiol.—American Journal of Pathology; Am. J. Physiol.—American Journal of Physiology; Appl. Environ. Microbiol.—Applied and Environmental Microbiology; FASEB—Federation of American Societies for Experimental Biology; JBC—Journal of Biological Chemistry; J. Physiol.—Journal of Physiology; Appl. Physiol. Nutr. Metab.—Applied Physiology, Nutrition, and Metabolism; Am. J. Pathol.—American Journal of Pathology; J. Agric. Food Chem—Journal of Agricultural and Food Chemistry; Int. J. Mol. Sci.—International Journal of Molecular Sciences; Food Chem.—Food Chemistry*.

Sixty-eight Science Daily posts refer to the scientific journal where the issue was published, four to the scientific event where the results of research about resveratrol were presented, and the focus of one post is based on research developed in a Ph.D. project. Four posts named the journal “Scientific Reports” (Impact Factor, IF 4.996) and three indicated the “PNAS” (IF 11.205) (Figure 3). Fifty-three posts indicate the DOI of the scientific paper. Eleven WebMD posts present information based on only one scientific paper. “*Thorax*” (IF 9.139) is the only journal mentioned twice (Figure 3). Two posts are about a topic presented in a scientific event and another two contain information provided by the University. WebMD publishes posts that are short reviews supported by different scientific references. We found nine posts of that type about resveratrol. WebMD does not indicate the DOI of the paper. In EurekAlert!, only one post refers to a scientific event. All the others mention the scientific journal (Figure 3). “*The American Journal of Pathology*” (IF 4.307) and “*Nature*” (IF 69.504) are mentioned two times each. Only seven posts indicate the DOI. None of the scientific journals mentioned in the posts are in the Scopus top five, which is composed of the “*Journal of Agricultural and Food Chemistry*” (IF 5.895); “*International Journal of Molecular Sciences*” (IF 5.542); “*Plos One*” (IF 3.24); “*Food Chemistry*” (266 papers) (IF 9.231); “*Nutrients*” (IF 5.429) (Figure 3). The five papers with the highest citation number in Scopus were not mentioned on any of the three online news portals. The paper from Howitz, K. T. et al., 2003 that occupies the Scopus sixth position, with 3086 citations, was mentioned by WebMD and EurekAlert!. We did not find any paper that was mentioned by the three online platforms (Figure 3). These results show the necessity to alert the newsrooms of the importance of providing scientific data regarding the post content. This information is important to increase the post’s credibility and to allow the reader to deepen their knowledge of the subject. Posts published in peer-reviewed journals generate more posts and, although there is no pattern, there seems to be a tendency to publish news about articles published in journals with the highest impact factor. These results also show that the papers that deserve more attention by researchers are not, necessarily, the ones that gain more attention from the media (Figure 2).

Some posts indicate the name of the scientific paper authors, mainly the first and last ones, as shown in Table 1. David Sinclair is the only author mentioned twice in Science Daily. In WebMD, we did not find the same author named more than once. Two posts on EurekAlert! also indicate David Sinclair and another two Stuart Richer. In Scopus, the authors’ top five papers with the higher number of publications about resveratrol are Das, D.K. (78 papers), Iinuma, M. (77 papers), Ito, T. (62 papers), Pezzuto, J.M. (60 papers), and Delmas, D. (57). In this list, David Sinclair appears in the thirty-three position. The fact that David Sinclair is the author that received more media attention could be explained by the “Sinclair Effect” previously mentioned. Again, it seems that the scientists that published more papers are not the ones that receive more attention from the media or maybe are the ones who think that is not important to promote their research through the media. Usually, the last authors are the senior ones, which can explain that last authors are the most mentioned by online media.

Further, 90% of the Science Daily posts report beneficial effects from resveratrol, 6% harmful effects, and 4% no effect. Eighty-four percent of the posts mention wine in the text and the average number of times counted in each post was 5.06 ± 1.04 (Mean ± Mean confidence interval). Half of these posts have wine in the title and, among these, only two report harmful effects, namely: “Alcohol consumption increases rosacea risk in women” and “Healthy’ component of red wine, resveratrol, causes pancreatic abnormalities in fetuses”, all the other posts report beneficial effects. In WebMD, 93% and 7% reported health benefits and harmful effects, respectively. The two posts that report unhealthy effects are “Red Wine Compound May Not Help Healthy Women” and “Could Red Wine Supplement Block Exercise Benefits?”. This last one is the only post that reports resveratrol’s harmful effects that has wine in the title. Almost 96% of the posts mention wine in the text, 70% of which has wine in the title. The word wine is mentioned 11.19 ± 4.25 times in each post. Regarding EurekAlert!, only the post entitled “Resveratrol, found in red wine, worsens MS-like symptoms and neuropathology in mice” reports resveratrol’s unhealthy effects, all the others declare its benefits for human health. The word wine is mentioned in 78% of the posts and among these, 43% in the title. On average, the word wine appears 2.93 ± 1.10 times in each post. Headlines, such as “Red Wine’s Resveratrol May Help Battle Obesity”, “Red wine: Exercise in a bottle?” or “Drinking Red Wine May Slow Aging”, as examples, are used to attract the attention of visitors to their sites. These online news portals earn revenue from advertising, so we can speculate they implement this kind of headline to increase visitor traffic or pageviews (Figure 2). A high traffic result is important to earn the trust of companies who want to advertise on their site.

### 3.2. Scientific Evidence for and Against

Resveratrol became popular in 1991 in the “60 Minutes” CBS show where Drs. Michel de Lorgeril and Serge Renaud were interviewed about the study called the French Paradox. According to the study, French people had a relatively low incidence of coronary heart disease because of their habit of drinking red wine, which would theoretically inhibit lipid peroxidation. Following this, resveratrol earned immediate popularity and triggered a vast amount of news in the media. The scientific studies mentioned in the posts, analyzed herein, report a diversity of bioactivities and considerable potential health effects of resveratrol, including antioxidant, anti-inflammatory, anti-obesity, anti-aging, chemopreventive, cardiovascular protective, or calorie-restriction mimicking effects.

After searching the scientific literature, it should be emphasized that the negative or non-positive affirmations described in the previous section come mainly from in vitro or animal studies and, consequently, the results cannot be directly extrapolated to human beings due to several limitations, such as differences in metabolism. Some scientific papers report that wine is a relevant contributor to resveratrol intake. Nevertheless, it is important to point out that, to obtain a quantity of resveratrol high enough to induce positive effects on health, in some cases, the amount of wine needed to be consumed exceeds what is considered “responsible” consumption, that is, regular and light to moderate consumption with meals.

Regarding the suggested pancreatic abnormalities in fetuses caused by resveratrol administration, only in one pre-clinical study in the literature was this effect found [44]. In non-human primates, Roberts et al., 2014 studied the effects of resveratrol supplementation, 3 months before breeding period and during the whole gestation period (0.37% w/w), in female Japanese macaques (4–7 years) fed a western diet (36% calories as fat). Indeed, after the nutritional intervention, resveratrol administered during pregnancy yielded improvements in maternal and placental phenotype, including beneficial effects in the fetal liver. However, an unexplained alteration in fetal pancreatic development was observed, characterized by an enlargement of the pancreas. Consistent with this finding, the authors also detected an increase in the proliferation marker Ki67 throughout the pancreas. This fact led the authors to suggest some advice against the use of resveratrol for pregnant women [44].

The statement “Red Wine Compound May Not Help Healthy Women” refers to a study reported by Yoshino et al., 2012 [45] from Washington University School of Medicine in St. Louis. In this study, healthy women in their late 50s and early 60s received resveratrol supplements (75 mg of *trans*-resveratrol) or placebo for 12 weeks, showing no improvement in factors linked to developing diabetes and heart disease. Subjects were non-obese (lean or overweight) postmenopausal women with no history or evidence of type 2 diabetes or cardiovascular disease and, therefore, it was difficult to find any effect on its putative molecular targets in skeletal muscle and adipose tissue or any improvement in metabolic function. Therefore, the lack of effect observed in this study is due to the fact that the individuals were healthy women and an improvement is unlikely. Consequently, this cannot be considered as a negative effect of resveratrol intake. In any case, other authors did find a decrease in serum total cholesterol values [46] or a small increase [47] after nutritional interventions, even in healthy subjects, but it should be emphasized that the data, before and after resveratrol intervention, remained always within a range accepted as physiologic (200–220 mg/dL).

As far as the effects of resveratrol supplementation on physical performance are concerned, they have been reviewed elsewhere [48,49,50,51]. Concerning the above-mentioned deleterious effects of resveratrol consumption on physical performance, the scientific evidence is scarce. Only in a pre-clinical study authored by Da Fonseca et al., 2021, an intriguing pro-oxidative state was observed [52]. In this study, Wistar rats were fed a high-fat diet and supplemented with resveratrol-rich beverages (resveratrol solution, whole grape juice, or red wine) combined with a running protocol, in training sessions lasting 10 min every day for 60 days. None of the experimental groups showed a deleterious effect related to resveratrol administration. Indeed, the unique negative result observed was an increased lipid peroxidation, measured as malondialdehyde (MDA) production, following the thiobarbituric acid method (TBARS), after the consumption of red wine, probably due to its alcoholic component and the alcohol-mediated reactive oxygen species (ROS) elevation during its metabolization [53]. Concerning other parameters determined, a significant increase in lean body mass or microbiota diversity and significant decreases in systolic blood pressure, interleukin-6, or lipid peroxidation values were observed in the whole-grape-juice-offered group, with no changes in resveratrol (15 mL/day of resveratrol solution 4%).

Contrarily, other pre-clinical [54,55,56,57,58] or clinical studies [59,60] did not find any deleterious effect of resveratrol supplementation on exercise performance, independently of its effect as an ergogenic aid. Thus, in a dose-dependent manner, the administration of resveratrol in combination with several training programs (plyometric exercise, high-intensity cycling, resistance training, …) shows positive effects, such as a reduction in the decline in strength performance [59], a reduction in pain or damage indexes [59], a reduction in the inflammation induced by plyometric-exercise-induced muscle damage [59] or exercise-induced inflammation [60], or fatigue [56,58] markers, and it could accelerate recovery [59] or increase grip strength [55,56,58], contractile force [54], or aerobic performance [55].

Regarding the effects of resveratrol administration on several symptoms of multiple sclerosis (MS), a disease characterized by demyelination and neuroaxonal damage that leads to the formation of lesions throughout the central nervous system, Sato et al., 2013 [61] suggested that resveratrol might have detrimental effects in some aspects of the disease. The authors studied the reported neuroprotective effects associated with sirtuin-1 activation by resveratrol. However, they found that resveratrol treatment significantly exacerbated demyelination and inflammation without neuroprotection in the central nervous system in two animal models (mice) of multiple sclerosis: experimental autoimmune encephalomyelitis (EAE) and Theiler’s murine encephalomyelitis virus-induced demyelinating disease (TMEV-IDD). Contrarily, a recent review [62] points out resveratrol as a possible intervention to regulate redox imbalance in microglia, which plays a critical role in neuroinflammation and neurodegeneration and, consequently, in neurological diseases. Considering these controversial statements, further research is warranted on this issue.

## 4. Conclusions

People today are exposed to multiple forms of digital media. As the digital media environment evolves, it is important to understand how news is produced, namely the ones that could influence people’s choices regarding their health. Due to the connection between resveratrol and wine consumption, in this study, we analyzed the resveratrol posts published in the digital news between 1990 and 2020, to cover the 30 years since the French Paradox study was published.

We observed that a higher number of posts was published in the years in which the literature points out as the beginning of the intensive use of the internet by people to seek information about health, diet, and nutrition. We also found that David Sinclair’s notoriety could explain the high number of posts between 2013 and 2018. The “Sinclair effect”, as we called it, emphasizes the importance of scientists communicating with the public and not just with their peers and the importance of doing so through the means that the public commonly uses, such as digital media.

According to this study, universities’ press releases are the main source of the news about resveratrol published in the digital media. This is in accordance with previous reports that most online journals chose to publish news stories, largely or entirely based on press releases from academic and research centers [63]. This can be considered a problem because the posts were, in most cases, a replication of the press releases.

This practice leads to the loss of the research function of journalism, which must be critical and vigilant of the public interest. This result alerts us to the importance of having science journalists in newsrooms, skilled in evaluating universities’ press releases critically and impartially.

The post-scientific background (name of the papers, authors, affiliation, journal title, DOI) depends on the editorial policy. The inclusion of these data is of crucial importance because it increases the post’s credibility and allows the reader to look for more information. Most of the posts analyzed reported results of preclinical studies, which highlighted the need for more research, especially involving cohort and clinical trials. Regarding the relationship between resveratrol and wine, the doses of resveratrol used are unlikely to be provided by wine in the frame of “responsible” consumption. Despite that, wine was commonly mentioned in the posts and their titles. Wine is used in a post title in a way that is intended to provoke public interest or excitement; however, these headlines are incongruent, since they do not accurately represent the information contained in the post. In our opinion, digital media must avoid attractive and profitable titles that do not mirror scientific results, particularly when these titles could influence people’s behavior with consequences for their health.

This is the first study reporting on the representation of the relationship between resveratrol and wine in digital news. A limitation of the current study is that it is a retrospective qualitative content analysis of online news, and the results obtained may or may not be like those obtained by analyzing other journalistic sources. Thus, the triangulation method is required for collecting more robust data. Nevertheless, these results are a new overview of the relationship between resveratrol and wine in online news and form a good basis for improving science communication, which should be audience centered. Additional research is necessary to establish more exhaustive and reproducible measures of misleading headlines over health biomedical science. The study of factors contributing to those headlines, particularly at the cultural and social levels of research, is needed to develop ways of reducing this practice. Researchers and journalists should be aware of ways to avoid it to guarantee precise research interpretation and publication. In conclusion, this report shows that scientists and scientific institutions intensify their efforts to communicate with the public to increase people’s health literacy. Scientists must have training in science communication. It also suggests that digital media should have journalists skilled in exploring scientific data and their translation into a simple and accurate language.

## Figures and Tables

**Table 1:** Authors of scientific papers cited in online posts.

Scientific Paper Authors	Science Daily	WebMD	EurekAlert!	Total
First	11	3	3	17
Middle (all or some)	2	2	2	6
Last	39	2	5	46
First and Middle (some)	0	0	0	0
Middle (some) and Last	4	0	2	6
First, Middle (some) and Last	4	0	0	4
First and Last	9	0	2	11
All authors	3	0	4	7
The person(s) mentioned is (are) not authors	5	11	0	16
No one is mentioned	2	19	0	21
Total of Posts	79	37	18	

## Data Availability

Not applicable.
